# Still in Plight: Traumatic Injuries and Their Acute Health Effects in Karachi, Pakistan

**DOI:** 10.7759/cureus.49956

**Published:** 2023-12-05

**Authors:** Saima Mushtaq, Muhammad Tarish Abro, Syeda Sakina Zehra

**Affiliations:** 1 Emergency Center, Jinnah Post Graduate Medical Center, Karachi, PAK; 2 Department of Medicine, Jinnah Sindh Medical University, Karachi, PAK; 3 Department of Medicine, Karachi Medical and Dental College, Karachi, PAK

**Keywords:** nature of injury, injury severity, road traffic accidents, emergency department, traumatic injuries

## Abstract

Introduction

Traumatic injuries are a leading cause of mortality and morbidity, with significant social and economic impacts. Karachi, Pakistan, a densely populated city with a high incidence of traumatic injuries, faces inadequacies in its trauma-care setup, including a lack of resources and unorganized care, which highlights the need for improved trauma management strategies and trauma registries. The purpose of this research is to present a fundamental profile of traumatic injuries in terms of their health-related consequences in Karachi, Pakistan. The objective is to identify the nature, affected body parts, and severity of traumatic injuries to identify areas for effective safety measures and reduce morbidity and mortality.

Methodology

This descriptive, cross-sectional study was conducted at Jinnah Postgraduate Medical Center (JPMC) in Karachi, Pakistan, from June to August 2021. The study included trauma patients over the age of 18 who presented at the accident and emergency department (ED) of JPMC during the study period. Data were collected using a structured questionnaire, and statistical analysis was performed using IBM SPSS Statistics. The study aimed to identify the demographic and clinical characteristics of trauma patients presenting to the ED.

Results

This study of 363 trauma patients found road traffic injuries (62.4%) as the most common mechanism of injury, with extremities (48.5%) being the most affected body part. The majority of trauma victims did not receive prehospital care (65.3%), highlighting a need for improved emergency response systems and public awareness. Open wounds (41.0%) were the most common nature of injury, with falls being the leading mechanism of fractures. The severity of injuries was mostly moderate (48.2%).

Conclusion

This study highlighted the need for targeted prevention strategies to reduce the health-related burden on the population of Karachi, Pakistan. Future research should focus on longitudinally monitoring all injured patients to identify high-risk populations, injury patterns, and preventative outcomes.

## Introduction

A traumatic injury can be defined as any physical injury to any living being affected by the surrounding conditions or extrinsic agents that necessarily require medical attention. As per the World Health Organization's (WHO) statistics, injuries account for an estimated 5.8 million fatalities each year. In particular, traumatic incidents stand tall as the primary cause of death prevalent among people aged between five and forty-four years (WHO, 2021) [1]. People who have experienced traumatic injuries often bear devastating consequences, including loss of life, mild to severe short-term disabilities, and long-term morbidities, as well as appreciable impacts on their social and economic lives due to lost productivity [2]. Moreover, there are effects on the mental health of the patient and their loved ones, such as post-traumatic stress disorder (PTSD), anxiety, and depression [3]. On the other hand, the different kinds of trauma also destroy property and hamper national economies by costing a huge sum in the management of the patients [4].

The emergency department (ED) of tertiary care hospitals plays a crucial role in the initial management of traumatic injuries [5]. Tertiary care hospitals are equipped with specialized medical staff and advanced technologies necessary for managing complex cases of traumatic injuries. Furthermore, these hospitals often serve as referral centers for patients with severe injuries who require specialized care.

Karachi, a metropolis with a human population of approximately 14,910,352 and a population density of 3,900 individuals per square kilometer (10,000/sq mi) [6], is called “the city that never sleeps” due to its all-time hustle and fast-paced life. Data shows that the incidence of traumatic injuries poses a significant threat in a bustling city like Karachi [7]. Despite trauma being a great menace, there are major inadequacies in the trauma-care setup in Pakistan. It includes a lack of medical resources and medical and paramedical personnel [7], unequipped ambulances [8], and unorganized pre-hospital and hospital-based trauma care [9]. The absence of trauma registries is another worth-mentioning issue because trauma registries, by documenting the acute care given to patients suffering from traumatic injuries, may contribute to improving its management through accountability and quality [8,10].

The prevention of injuries in developing countries is a crucial aspect that requires urgent attention. Pakistan is one such nation that is grappling with the challenge of increasing injury rates. In this context, there is a pressing need for policy initiatives that can address the root causes of injuries and provide effective solutions. The purpose of this research is to present a fundamental profile of traumatic injuries and to understand the burden of traumatic injuries in terms of their health-related consequences in Karachi, Pakistan. After conducting a thorough review of existing literature, it has been determined that there is currently no research article available that specifically examines the nature of injury, the body parts that are typically affected, and the severity of these injuries within the particular setting under investigation, indicating a significant gap in the current knowledge and understanding of injury patterns in this context. Hence, finding out areas where efforts can be made to drive maximum benefit for safety against morbidity and mortality due to traumatic events is important. The research objectives were to identify the various types of traumatic injuries, determine the body parts most affected by traumatic events, and assess the severity of these injuries in Karachi, Pakistan.

## Materials and methods

Study design and setting

This was an observational descriptive study with a cross-sectional approach, which was conducted in the accident and emergency department of Jinnah Postgraduate Medical Center (JPMC), Karachi, Sindh, Pakistan, from June 2021 to August 2021. JPMC was selected as the setting because it is one of the largest tertiary care hospitals in Karachi, Pakistan, and a majority of injured patients are referred to JPMC’s emergency ward every day either for initial treatment or for onward referral to specialized wards due to all kinds of traumatic events, including road traffic accidents (RTAs; traumatic accidents involving any vehicle), physical violence, firearms, falls, locomotive accidents, etc.

Participants

Trauma patients, according to the definition of trauma given above, irrespective of sex, religion, class, color, or ethnicity, and above the age of 18 years, who presented for the first time to the ED of JPMC throughout the study period, were included. Those patients who were brought dead to the hospital or died immediately upon arrival, were unconscious, unidentified, or unaccompanied, were excluded from the study.

Sample size

According to the inclusion criteria, the population size of patients presenting to the ED in three months was estimated to be around 24,000. Hence, using the open EPI Info software, the sample size was calculated to be 379. The margin of error for the sample size calculation was kept at 5%, and a confidence interval of 95% was set. Non-probability convenience sampling was done.

Data collection and variables

After getting approval from JPMC’s ethics review board to conduct the research and obtaining informed written consent from the patients or their attendants in case the patient was not able to provide consent, data were recorded from all the included cases (IRB approval NO.F.2-81/2021-GENL/56927/JPMC). Data were collected by medical students and doctors using a structured, standardized questionnaire that was made using the injury surveillance guidelines provided by WHO (WHO/NMH/VIP/01.02). The questionnaire included variables such as patient demographics, including sex, age, literacy (patients were classified as literate if they attended any educational institute and as illiterate if they never did); employment (employed refers to a person who is currently working for pay or profit, either as an employee or self-employed and unemployed refers to a person who is willing and able to work but is currently without a job or employment); mechanism of injury; road-traffic variables such as mode of travel, type of road user, and whether the patient was wearing a seatbelt and a helmet (because road-traffic accidents have been believed to be the most frequent cause of trauma); intent of the injury (classified as intentional when an assault was carried out by some other person, unintentional, and self-harm). The severity and nature of the injury, as well as the body part injured, were also recorded and documented. The severity of injuries was evaluated through physical examination and classified based on the same WHO injury surveillance guidelines into three categories: minor (such as bruises and minor cuts), moderate (such as fractures and sutures), and severe (such as internal hemorrhage and punctured organs).

Statistical analysis

The Kolmogorov-Smirnov test was run to determine the normality of the distribution of continuous variables. Continuous, normally distributed variables were expressed as means ± standard deviations. Interquartile ranges (IQRs) and medians were calculated for continuous, non-normally distributed variables. The main categorical variables, such as the injured parts of the human body, the nature of injuries, and the severity of injuries, were presented by their frequencies and percentages. We used IBM SPSS Statistics for Windows, Version 25 (released 2017; IBM Corp., Armonk, New York, United States) for statistical analysis. We further stratified our data to check for confounders and noted the frequencies and percentages of injuries in different parts of the human body, the different natures of injuries, and the severities of injury for the patient demographics (sex, age group, literacy, and occupation), the mechanisms of injury, the intents of injury, and hemodynamic stability of the patients post-trauma. Different injured parts of the body were also cross-analyzed with their severities. We cross-analyzed the different natures of injuries with the injured parts of the body. Moreover, the severity of the injury was cross-analyzed with the nature of the injury. For the patients injured by road traffic accidents, we stratified our data based on the mode of transport, type of road user, use of a helmet, use of a seatbelt, as well as the different injured parts of the body, nature, and severity of injury. As this was a descriptive study, we did not conduct significance testing.

## Results

Demographic characteristics

Data were collected from a sample of 379 trauma patients. Since no imputation methods were used, after excluding those responses with missing data, the final total number of responses obtained was 363. The male-to-female ratio was 4.3:1 (see Table 1). The age range of the participants was 18 to 85 years, and we divided it into age groups, i.e., 18-25: young adults (27.9%), 26-35: adults (30.1%), 36-45: middle-aged (20.4%), and >46: aged (21.5%). The mean age of the patients was 35.8 years old (SD ± 13.9) (95% confidence interval (CI) 34.4-37.2).

**Table 1:** Patient demographic characteristics CI: confidence interval

	Frequency (n)	Percentage (%)	95% CI
Gender			
Male	295/363	81.3%	77.3% - 85.3%
Female	68/363	18.7%	14.7% - 22.7%
Literacy			
Literate	235/363	64.7%	59.8% - 69.7%
Educated till primary plus secondary school	138/233	59.2%	54.2% - 64.3%
Employment			
Employed	237/363	65.3%	60.4% - 70.2%
Mechanism of injury			
Road traffic injury	226/363	62.4%	57.4% - 67.4%
Fall	49/363	13.5%	10.0% - 17.1%
Other blunt forces	34/363	9.4%	6.4% - 12.4%
Struck/hit by a person (assault)	23/363	6.4%	3.8% - 8.9%
Cut/stab	16/363	4.4%	2.3% - 6.5%
Gun shot	14/363	3.9%	1.9% - 5.9%
Road user			
Drivers	120/220	54.5%	49.4% - 59.7%
Seatbelts			
Not used	15/18	83.3%	79.5% - 87.2%
Helmets			
Not worn	118/157	75.2%	70.7% - 79.6%
Brought by			
Ambulance	148/363	40.8%	35.7% - 45.8%
Duration in reaching to the hospital			
Four to 24 hours	62/363	17.1%	13.2% - 21.0%
Pre-hospital care			
No	237/363	65.3%	60.4% - 70.2%

A majority of the participants (64.7%) reported being literate, and a significant portion of them (59.2%) had not received higher education beyond the secondary school level. The data suggests that a majority of the population (65.3%) is employed, indicating their participation in the workforce.

The data shows that road traffic injuries were the most common mechanism of injury (62.4%), followed by falls (13.5%), blunt forces (9.4%), assault (6.4%), stab injuries (4.4%), and gun-shot injuries (3.9%). Drivers represented the largest proportion of road users who suffered injuries, at 54.5%. A large majority of trauma victims did not use seatbelts (83.3%) or helmets (75.2%).

A little over a third of trauma victims, at 40.8%, were brought to the hospital by ambulance, and a notable proportion, at 17.1%, took four to 24 hours to reach the hospital after an injury, potentially indicating a need for improved emergency response systems and public awareness regarding the importance of timely medical care for trauma cases.

Parts of the body most affected by traumatic injuries

**Figure 1:**
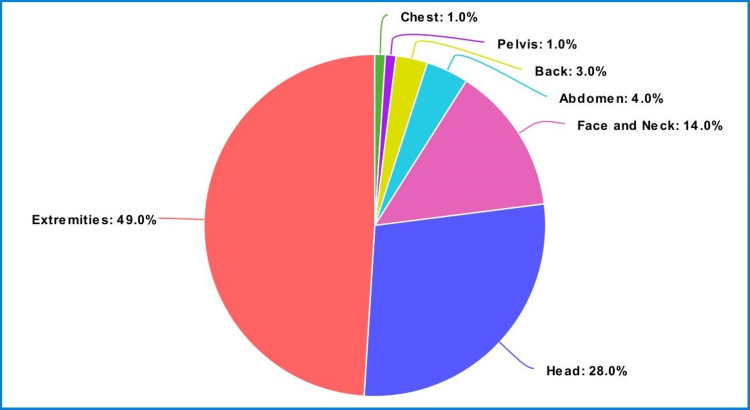
Pie-chart for the parts of the body most affected by traumatic injuries

Figure 1 and Table 2 show that we observed traumatic injuries in descending order: extremities (48.5%), head (28.1%), face and neck (14.2%), abdomen (3.8%), back (3.4%), chest (1.3%), and pelvis (0.7%).

**Table 2:** Parts of the body injured

Parts of the body injured	Frequency (n)	Percentage (%)	95% CI
Abdomen	17/445	3.8%	1.8% - 5.8%
Back	15/445	3.4%	1.5% - 5.2%
Head	125/445	28.1%	23.5% - 32.7%
Chest	6/445	1.3%	0.2% - 2.5%
Extremities	216/445	48.5%	43.4% - 53.7%
Face and neck	63/445	14.2%	10.6% - 17.7%
Pelvis	3/445	0.7%	-0.2% - 1.5%

A comparable proportion of males and females, with 49% (146/295, 95% CI 43.9%-54.1%) and 47% (32/68, 95% CI 41.9%-52.1%) respectively, suffered injuries to extremities. Hence, gender may not be a significant predictor of this type of injury among trauma victims. This was also true for age groups, literacy, and employment. However, two-thirds of pelvic injuries (2/3 (66.7%), 95% CI 61.8%-71.5%) were suffered by housewives/househusbands.

A substantial proportion of injuries, at 43.8% (159/363, 95% CI 38.7%-48.9%), were due to motorcycle accidents, with patients suffering injuries to all body parts except for the pelvis. Conversely, all pelvic injuries were suffered by pedestrians (3/3, 100%).

Even though a significant majority of extremity injuries, at 91.6% (163/178, 95% CI 88.7%-94.4%), were unintentional, two-thirds of self-harm injuries were also to the extremities (2/3 (66.7%), 95% CI 61.8%-71.5%).

Nature of traumatic injuries

**Figure 2:**
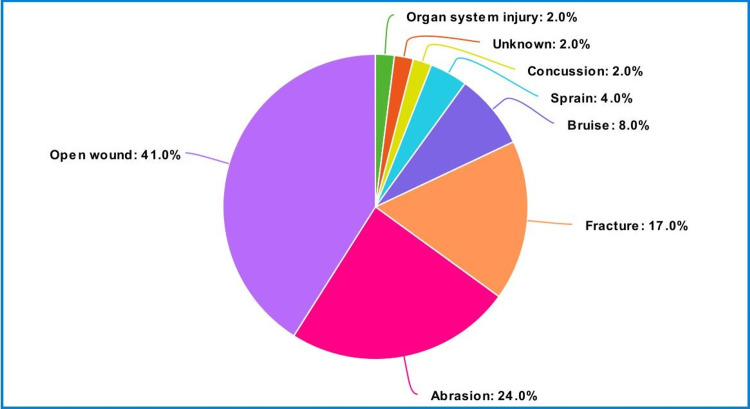
Pie-chart for nature of traumatic injuries

Figure 2 and Table 3 display the injuries sustained by the participants in descending order, including open wounds (41.0%), abrasions (23.9%), fractures (17.5%), bruises (7.8%), sprains (3.9%), concussions (2.0%), and organ system injuries (2.0%).

**Table 3:** Nature of traumatic injuries CI: confidence interval

Nature of injury	Frequency (n)	Percentage (%)	95% CI
Abrasion	122/510	23.9%	19.5% - 28.3%
Bruise	40/510	7.8%	5.1% - 10.6%
Concussion	10/510	2.0%	0.5% - 3.4%
Fracture	89/510	17.5%	13.5% - 21.4%
Open wound	209/510	41.0%	35.9% - 46.0%
Organ system injury	10/510	2.0%	0.5% - 3.4%
Sprain	20/510	3.9%	1.9% - 5.9%
Unknown	10/510	2.0%	0.5% - 3.4%

A larger proportion of males (104/295 (35.3%), 95% CI 30.3%-40.2%) than females (16/68 (23.5%), 95% CI 19.2%-27.9%) suffered from open wounds. Age group, literacy, and occupation were not significant predictors of the nature of traumatic injuries suffered by the population.

Falls were the most common (18/34 (52.9%), 95% CI 47.8%-58.1%) mechanism of injury leading to fractures. Self-harm injuries were most commonly (2/3 (66.7%), 95% CI 61.8%-71.5%) inflicted by open wounds. The majority (2/3 (66.7%), 95% CI 61.8%-71.5%) of patients who suffered pelvic injuries experienced a range of traumatic injuries, including fractures, open wounds, and bruises.

Severity of the traumatic injuries

**Figure 3:**
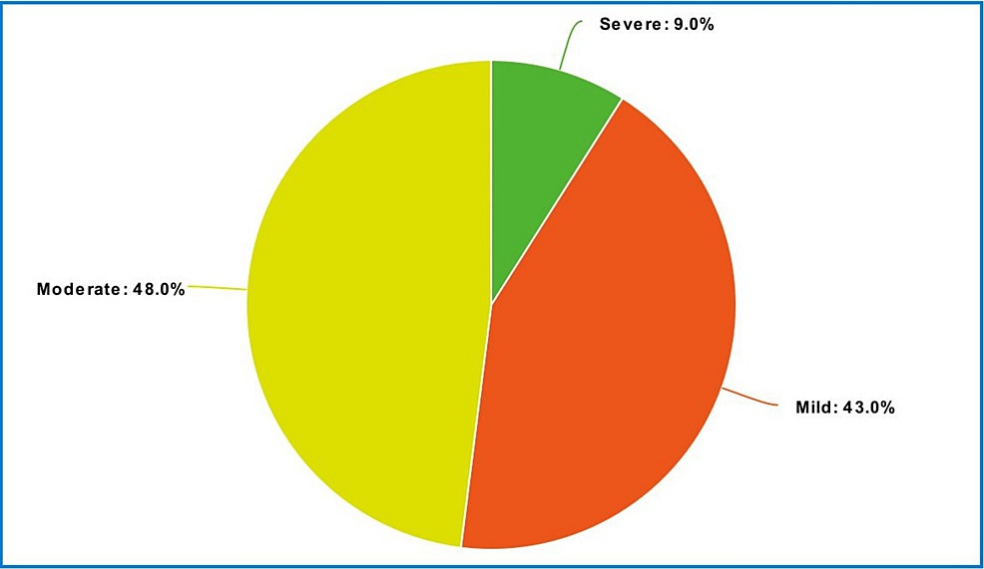
Pie-chart for the severity of the traumatic injuries

Figure 3 and Table 4 show that our sample manifested traumatic injuries that were, in descending order, moderate (48.2%), mild (43.0%), and severe (8.8%).

**Table 4:** Severity of the traumatic injuries CI: confidence interval

Severity	Frequency (n)	Percentage (%)	95% CI
Severe	32/363	8.8%	5.9% - 11.7%
Moderate	175/363	48.2%	43.1% - 53.3%
Mild	156/363	43.0%	37.9% - 48.1%

There was no significant difference in the frequency of mild, moderate, and severe injuries between males and females in the trauma victim population. The same was true for literacy. Young adults (50/156 (32.1%), 95% CI 27.3%-36.9%) may be more susceptible to mild injuries than adults (48/156 (30.8%), 95% CI 26.0%-35.5%) despite their lower representation in the sample. Higher education levels, such as college (23/46 (50.0%), 95% CI 44.9%-55.1%) and university (26/49 (53.1%), 95% CI 47.9%-58.2%), may be associated with a higher likelihood of suffering mild injuries rather than moderate injuries.

There was a higher frequency of mild injuries among housewives/househusbands (17/31 (54.8%), 95% CI 49.7%-60.0%), and students (10/19 (52.6%), 95% CI 47.5%-57.8%). Injuries resulting from self-harm were evenly distributed across the mild, moderate, and severe categories. A majority of moderate-severity injuries in the sample population were unintentional (153/175 (87.4%), 95% CI 84.0%-90.8%).

All fractures (34/34, 100%) in the sample population were of moderate severity. Approximately one-third (10/32 (31.3%), 95% CI 26.5%-36.0%) of severe injuries were attributed to open wounds. Two-thirds (2/3 (66.7%), 95% CI 61.8%-71.5%) of patients who sustained injuries to the pelvis had severe injuries.

## Discussion

Traumatic injuries are one of the leading causes of death and disability worldwide [11]. Our study provided insight into the health-related burden inflicted by trauma on individuals aged 18 years and older. Since 62.4% of our sample had been injured due to road traffic accidents and provided that in Pakistani and Muslim culture, men customarily go out to serve as the breadwinners for their families, we speculate that this led to the greater percentage of men suffering from injuries in our sample, i.e., 81.3%, as compared to that of women, i.e., 18.7%. Similar results were found in other studies as well, from Pakistan, Tehran, and Shiraz, respectively [7,9,10]. According to our findings, the age group of 25 to 35 years old was the most vulnerable to traumatic injuries, possibly because of the optimum activity levels in this age group. A study by Fararoei et al. also showed the same result in Yasuj, Iran [12]. RTAs were also found to be highly prevalent in other centers in Pakistan, thus making RTAs a serious public health concern [13,14]. In the context of Karachi, this could be true because Karachi is the most populated city in Pakistan, and as the population grows, so does the number of automobiles on the road. Due to the lack of effective rail and urban transport, almost all passenger and goods transport goes by road [15].

One-third of the patients were transported to the hospital by ambulance, while 17.1% arrived at the facility four to 24 hours after sustaining the injury. Peyravi et al. conducted a study that showed that short-term placement of ambulance units significantly decreased response times and improved the initial survival rates of patients [16]. In our scenario, few patients received prehospital treatment, possibly due to Pakistan's underdeveloped ambulance system [17]. Moreover, the time taken to reach a hospital can have a significant impact on the outcomes of patients who have sustained injuries. This is because certain injuries require urgent medical attention, and the longer it takes for a patient to receive treatment, the greater the risk of complications and long-term consequences.

Our study showed that the majority of the traumatic injuries sustained were to the extremities. The possible reason for this may be the high mobility of the extremities compared to other parts of the body. Moreover, it is a common reflex to utilize one's extremities to protect oneself from injury during a traumatic event. Injuries to the extremities can cause significant pain, swelling, and loss of function, which can lead to temporary or permanent disability. In some cases, traumatic injuries to the extremities can also result in complications such as infections, nerve damage, and blood clots. A study conducted in the United Kingdom showed that a majority of injuries were to the upper extremities [18].

Our findings showed that the most prevalent type of injury was open wounds, followed by abrasions and fractures. This showed that the penetrating types of trauma were more dominant than the blunt types of trauma. This result was inconsistent with some studies, in which blunt trauma outnumbered penetrating ones [19,20]. The higher incidence of penetrating trauma injuries in the setting of Karachi could be due to a combination of various factors, including the prevalence of road traffic accidents, violence, and cultural and societal norms. Falls were the most prevalent mechanism of injury resulting in a fracture. This is because of the force and impact generated when the body hits a hard surface. In addition, falls can occur in a variety of settings, such as at home, in the workplace, or during sports or recreational activities, which may increase the likelihood of sustaining a fracture. This result can help in the development of targeted prevention strategies that address the specific risk factors associated with different types of injuries.

Almost half, i.e., 48.2%, of the presenting traumatic injuries, were of moderate severity. The results also showed that, compared to adults who suffered more from moderate injuries, most young adults suffered from mild injuries. This may be due to factors such as better bone and muscle strength and a lower risk of underlying medical conditions. All fractures were of moderate severity because, in most cases, they can cause significant pain and discomfort but are not typically life-threatening. Open wounds were attributed to the greatest number of severe injuries, according to our findings. This might be due to factors such as increased risk of bleeding, longer healing time, and increased risk of infection.

The findings indicated that more than half of the individuals with traumatic injuries were employed. The connection between socioeconomic status and health had been well established, and lower socioeconomic positions were directly associated with negative post-injury outcomes such as anxiety, depression, worse functional outcomes, and long-term mortalities [21,22]. Additionally, trauma victims who were already struggling financially were shown to carry a greater risk of experiencing a loss of work and reduced incomes after their accidents [23].

Opportunities to prevent injuries occur through a range of educational approaches [24]. According to the findings, the majority of people who sustained injuries were literate. These findings may be useful in developing safety precautions and prevention strategies that finally improve the quality of life of the common people. Moreover, future research may include longitudinally monitoring all injured patients to identify high-risk populations, injury patterns, and preventative outcomes.

Limitations

Trauma patients below the age of 18 were missed in the study, which, if included, could have added to the knowledge of the health-related consequences of trauma.

We used convenience sampling as the sampling type. However since the sample size was huge and with a 95% confidence interval, we hope that our data proved to be important, while we agree that more stringent sampling types would confirm the results. In addition, the study collected limited morbidity data. Long-term disabilities could not be recorded because the study did not include follow-up. The study only mentions acute mortality. The study did not discuss three- to seven-day mortality, in-hospital mortality, or 30-day mortality as it falls outside the scope of this study. Finally, because this study was a single-centered one, more information needs to be collected from multiple trauma centers in Karachi to completely understand the threat of traumatic injuries in Karachi, Pakistan.

## Conclusions

Our study addressed the site, nature, and severity of traumatic injuries in Karachi, Pakistan. RTAs were the leading causes of injury. Extremities were the most common sites involved. Open wounds were the most prevalent type of injury, and the majority of injuries were of moderate severity. There is a need to address injury prevention and control in these areas. Future research may include longitudinally monitoring all injured patients to identify high-risk populations, injury patterns, and preventative outcomes.
